# Significant efficacy of tofacitinib from China in the treatment of alopecia areata: a case report

**DOI:** 10.3389/fimmu.2025.1553904

**Published:** 2025-04-11

**Authors:** Yijia He, Jinxu Qi, Zhuochen Wu, Jinfang Zhang, Guoqiang Zhang

**Affiliations:** ^1^ Department of Dermatology, The First Hospital of Hebei Medical University, Shijiazhuang, Hebei, China; ^2^ Subcenter of National Clinical Research Center for Skin and Immune Diseases, Shijiazhuang, Hebei, China; ^3^ Hebei Provincial Innovation Center of Dermatology and Medical Cosmetology Technology, Shijiazhuang, Hebei, China

**Keywords:** alopecia areata, JAK/STAT, JAK inhibitors, tofacitinib, treatment

## Abstract

Alopecia Areata (AA) is a common form of scarless alopecia. Its pathogenesis may be related to T cell-mediated autoimmune attack on hair follicles. Its clinical manifestations are mostly round or oval patches of AA, which can progress to Alopecia Totalis (AT) and Alopecia Universalis (AU). In severe cases, it affects the psychological health and quality of life of patients. In the past, the treatment of AA mainly relied on intra-lesional or systemic application of glucocorticoids, minoxidil or immunomodulators, which had problems such as limited efficacy and high recurrence rate. Some studies have also found that JAK inhibitors have improved effects on many autoimmune diseases, including AA. This case report presents a patient with AA who achieved significant therapeutic effects from treatment with Tofacitinib Citrate Sustained-Release Tablets, manufactured by Qilu Pharmaceutical Company in China. Due to the recurrence rate of AA, patients are likely to need long-term medication. The resulting economic burden cannot be ignored. We therefore investigated the mechanisms and economic benefits of various JAK inhibitors in the treatment of AA, in order to provide better guidance to patients with recurring disease who need long-term medication, and to their doctors in choosing a more rational therapeutic agent according to the patient’s condition and economic status.

## Introduction

Alopecia Areata (AA) is a common form of non-scarring alopecia that tends to present as round or oval patches of hair loss. As the disease progresses, it may lead to hair loss in localized scalp areas, the entire scalp (including eyelashes and eyebrows), or even the entire body (1). The pathogenesis may be associated with T-cell-mediated autoimmune attacks on hair follicles (2). Current first-line treatments for AA rely on intralesional or systemic corticosteroids, minoxidil, or immunomodulatory agents, which are limited in efficacy, have significant side effects, and high recurrence rates, especially for patients with severe AA (3).

Studies have indicated that Janus kinase (JAK) inhibitors, as a novel therapeutic target, can inhibit type 1 and 2 cytokines, thereby reducing the immune response and the associated inflammatory reactions (4). Conclusions have been drawn that tofacitinib appears to be a promising drug for the treatment of AA, with minimal side effects (5). Herein, we report a case of a patient with AA who achieved significant therapeutic effects from treatment with Tofacitinib Citrate Sustained-Release Tablets manufactured by Qilu Pharmaceutical Company in China (Chinese Tofacitinib), and discuss the mechanisms and efficacy of different JAK inhibitors in the treatment.

## Case presentation

**Figure 1:**
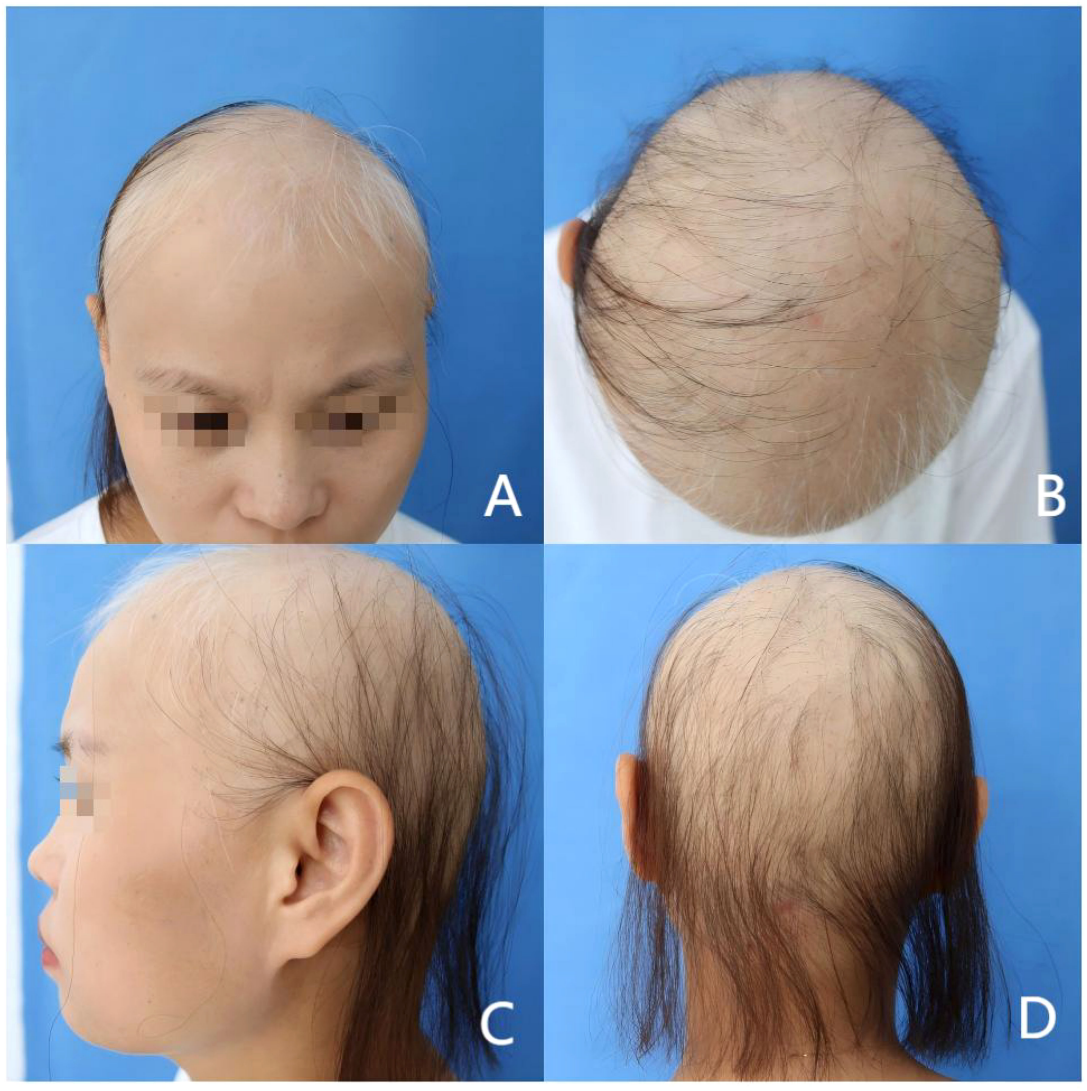
The patient has diffuse hair loss on the scalp and partial loss of eyebrows. SALT score was 72. **(A)** Front of the head; **(B)** Top of the head; **(C)** Side of the head; **(D)** Back of the head.

The patient is a 33-year-old female who presented with hair loss for half a year. Initially, she experienced patchy alopecia, which progressively worsened over the course of 2 months, manifesting as diffuse hair loss. She had previously attempted treatment with traditional Chinese medicine, which yielded disappointing results. Her urgent need for treatment led her to seek dermatological consultation. Physical examination revealed diffuse hair loss on the scalp with scattered, thin, and fine hairs, and partial eyebrow loss, as shown in (**Figure 1**). Trichoscopy identified typical yellow dots, black dots, and short vellus hairs. The patient’s serum IgE was elevated at 432 IU/ml (normal range 0.0-375.0 IU/ml), and cytokine levels were increased: IL-5: 29.27 pg/ml (normal range 0.00-3.10 pg/ml), IL-6: 34.61 pg/ml (normal range 0.00-7.00 pg/ml), IL-8: 1276.72 pg/ml (normal range 0.00-20.60 pg/ml). The patient was ultimately diagnosed with AA.

**Figure 2:**
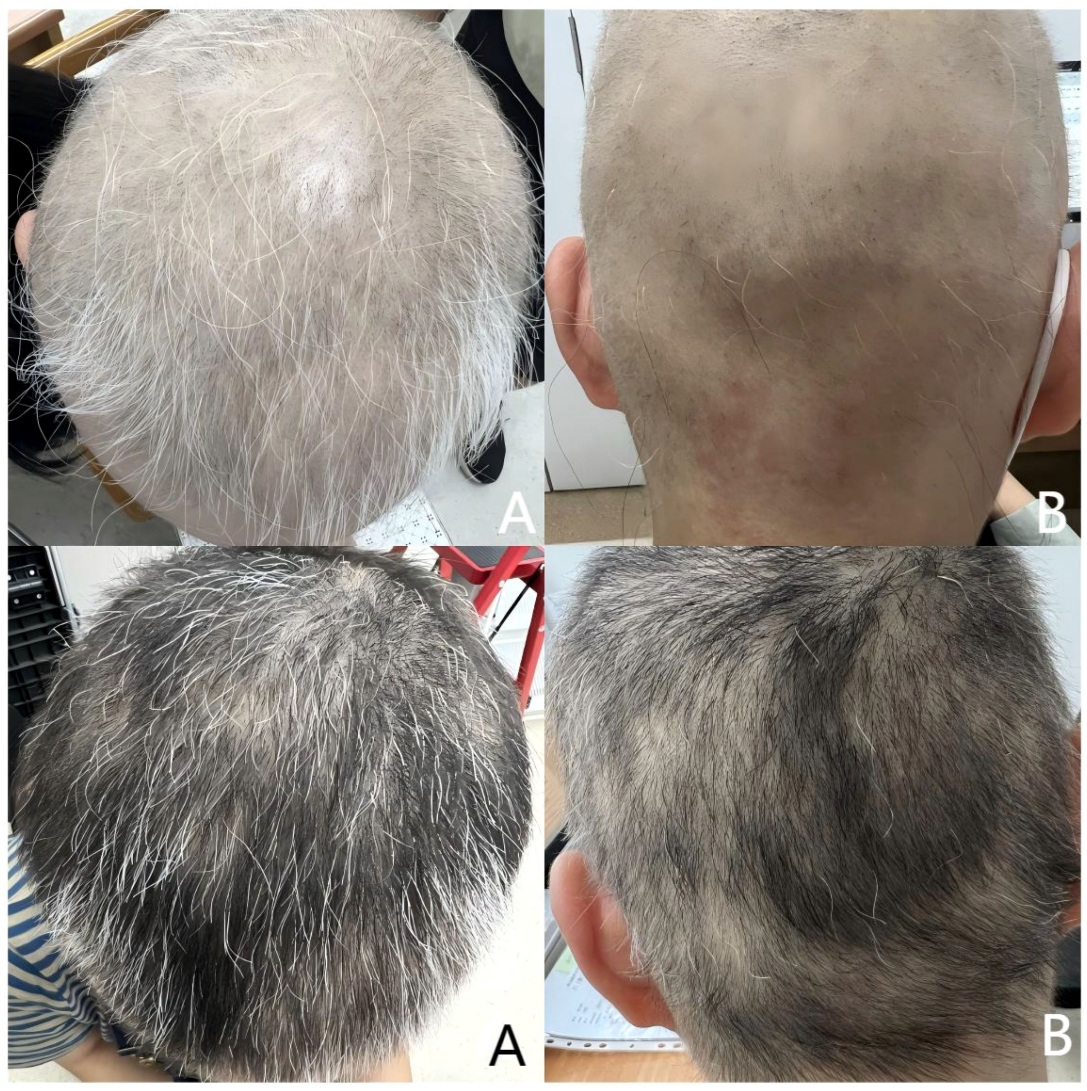
Upper half is after 2 months of treatment. SALT score was 66. Lower half is after 3 months of treatment. SALT score was 34. **(A)** Top of the head; **(B)** Back of the head.

**Figure 3:**
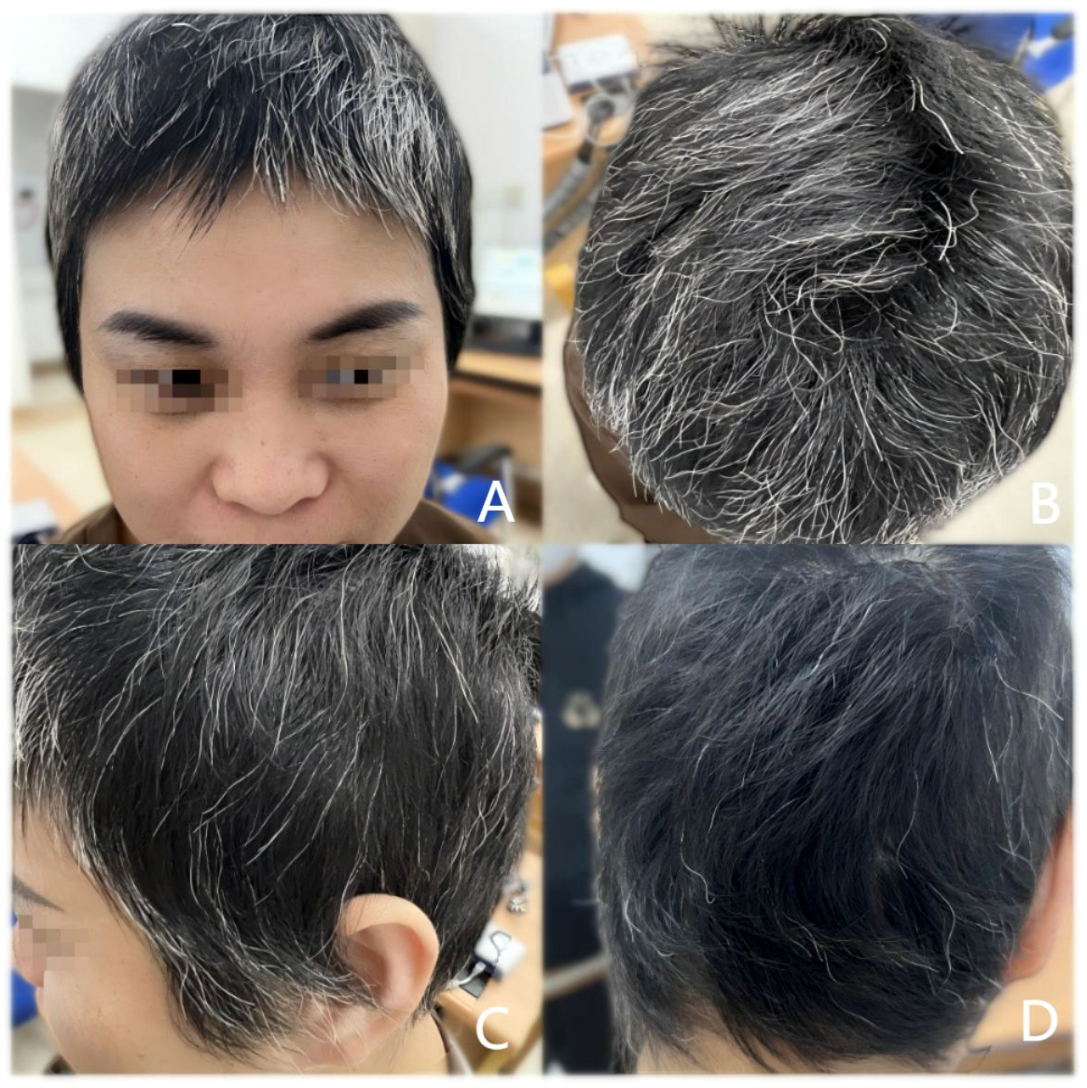
After 5 months of treatment. The patient’s scalp hair and eyebrows became thicker. SALT score was 12 **(A)** Front of the head; **(B)** Top of the head; **(C)** Side of the head; **(D)** Back of the head.

The patient was previously healthy, she denied a family history of AA. And there were no other autoimmune diseases and atopic diseases in her or her family. She was very anxious about her appearance and did not want to participate in social activities, which had seriously affected her normal work and life. After consultation, the patient consented to treatment with Chinese Tofacitinib. She was prescribed a dosage of 11mg once daily. We followed up with the patient at 2, 3, and 5 months post-treatment. Her scalp grew dense hair, and her eyebrows became thicker compared to before, as shown in **Figures 2** and **3**. Cytokine levels returned to normal. And before the treatment, her SALT score (represents the degree of percentage of hair loss on the scalp, higher scores mean more severe hair loss) was 72, and after 2, 3 and 5 months of treatment, her SALT scores were 66, 34 and 12, respectively. The patient was very satisfied with the treatment outcomes. After a total of 6 months of treatment, the patient discontinued the medication and experienced a relapse of hair loss patches one month after stopping the medication. Consequently, the patient resumed oral administration of Chinese Tofacitinib and has continued to date, with the alopecia patches have not reappeared.

## Discussion

AA is an autoimmune disease with T-cell-mediated damage to hair follicles (6). The periphery of lesioned hair follicles is infiltrated with inflammatory cells, resulting in stagnation of hair growth in the anagen phase, leading to non-scarring alopecia. It is characterized by small patches of alopecia in normal hair-bearing skin. Variants of AA include AT, characterized by complete absence of scalp hair, and AU, characterized by complete absence of scalp and other body hair. In more severe cases, the condition affects the patient’s mental health and quality of life, and even has a tendency to trigger depression. For mild cases of AA, local or intra-lesional steroid injections are the main first-line treatment, while for more severe cases, systemic application of immunosuppressants is the mainstay of treatment. However, severe AA is difficult to cure and has a high recurrence rate, and treatment remains a great challenge (7).

The etiology of alopecia areata (AA) is not yet fully understood, and it may be triggered by a combination of genetic predisposition, environmental factors, and immune responses. Some studies speculate that the collapse of immune privilege in hair follicles caused by IFN-γ produced by CD8+ T cells induces the production of IL-15 and promotes type I cell autoimmunity, thereby facilitating the onset of AA. By blocking the common signaling pathways downstream of cytokine receptors, especially the JAK/STAT pathway, AA can be reversed in mice (8). Consequently, JAK inhibitors have emerged as a new class of targeted drugs. In a study of 125 patients with alopecia areata, 83.2% improved after using tofacitinib and 16% achieved complete remission (9). Based on previous case reports, this patient ultimately chose to receive treatment with Chinese Tofacitinib as well.

Tofacitinib is a representative Janus kinase (JAK) inhibitor. It exhibits a stronger inhibitory effect on the JAK1 and JAK3 pathways, while its inhibitory effect on JAK2 is relatively weaker. It has been approved by the U.S. Food and Drug Administration (FDA) for the treatment of moderate to severe rheumatoid arthritis and ulcerative colitis. In the field of dermatology, results have shown that tofacitinib has certain therapeutic effects on psoriasis, alopecia areata, atopic dermatitis, and vitiligo (10). Common adverse effects include mild infections (upper respiratory tract infections), headache and gastrointestinal upset. In this case, the patient showed a good therapeutic response after using Chinese Tofacitinib, and no adverse reactions were observed.

Upadacitinib is an oral JAK1 inhibitor that has been found to have significant therapeutic effects in the treatment of rheumatoid arthritis, moderate to severe atopic dermatitis, and ankylosing spondylitis (11). Compared to first-generation drugs, Upadacitinib has a higher level of selective JAK1 inhibition and better safety. It may be a more optimal treatment option for AA (12). There are reports in the literature of using Upadacitinib to treat five cases of alopecia areata with different backgrounds and severities. The dosage used was 15 mg, once a day, and satisfactory therapeutic effects were achieved in all cases, with no adverse events reported (13). Currently, the use of Upadacitinib for the treatment of AA is still considered off-label. Although it has a rapid onset of action, potential adverse reactions, including infections, tuberculosis, and tumors, must be closely monitored. Furthermore, additional research is needed to evaluate its efficacy in treating AA, as well as the timing and methods for dose reduction and discontinuation.

Baricitinib is a selective JAK1 and JAK2 inhibitor that has shown efficacy in phase II and phase III randomized controlled trials for adult patients with severe AA (14). Additionally, it has been reported that Baricitinib demonstrates good therapeutic effects on AA, particularly in patchy AA (15). In two phase III trials, approximately 35-40% of patients achieved significant hair regrowth (SALT ≤ 20) at 52 weeks of treatment, and the response lasted up to 104 weeks (16). Currently, Baricitinib is already FDA approved for the treatment of severe alopecia areata in adults. However, common adverse effects include upper respiratory tract infection, acne, headache, dyslipidemia, and elevated creatine kinase (17). Discontinuation of the drug may lead to disease recurrence, and long-term maintenance therapy is required to consolidate the effect (18).

Abrocitinib is a highly selective JAK1 inhibitor that has been approved for the treatment of moderate to severe atopic dermatitis (abbreviated as AD). A case report described a patient with AU and AD. The patient developed an increase in drug eruption with eosinophilia and systemic symptoms (abbreviated as DRESS) after taking psychotropic medication, and two months later, developed total hair loss (diagnosed as an autoimmune sequela of DRESS). Initially, the patient was treated with tofacitinib at a dosage of 5mg once daily, but continued to experience hair loss after two months. The treatment was then switched to Abrocitinib at a dosage of 100mg once daily for two months, followed by an increase to 200mg once daily for an additional two months. After six months of treatment, the patient’s hair loss symptoms disappeared, and no significant adverse reactions were reported (19). In the short term, Abrocitinib appears to be more effective and faster-acting than tofacitinib for the treatment of AU (although it is possible that the duration of tofacitinib use was too short and the daily dosage was insufficient). It may also have certain therapeutic effects on AA.

Ritlecitinib is an oral, selective dual JAK3/TEC family kinase inhibitor. A clinical trial showed that patients treated with 50 mg or 30 mg Ritlecitinib achieved a SALT score of ≤20 at 24 weeks, respectively, significantly better than control group (2%) (20). It has a good overall safety profile, with common adverse effects including headaches, upper respiratory tract infections and acne (21). It may applicable to refractory cases that are ineffective or recurrent to traditional treatments (such as glucocorticoids and immunosuppressants).

Deuruxolitinib is an oral JAK1/JAK2 inhibitor. After 24 weeks of treatment with Deuruxolitinib 12 mg twice daily, 41.3% of patients achieved SALT ≤20, significantly higher than control group (0.8%), and it was better than Ritlecitinib 50 mg and Baricitinib 4 mg in patients with severe alopecia areata (highest SUCRA score) (22). However, it needs to wait for formal approval and more long-term safety data.

## Conclusion

AA is a common non-scarring hair loss condition, and current treatments are limited by their efficacy and high relapse rates. JAK inhibitors, as a new class of targeted drugs, show great potential. Different JAK inhibitors vary in mechanism, efficacy, and speed of onset. This case report describes a successful treatment of AA with Chinese Tofacitinib, ensuring its effectiveness and safety. However, this study is limited by a small sample size. The patient’s hair loss improved after half a year of medication but relapsed one month after discontinuation, and then continued to take the medication to date. This also indicates that treating AA is likely to be a long-term process, and the issues of continuous treatment and relapse after discontinuation need to be considered. More observations and research are needed in the future to fully assess the potential of various JAK inhibitors in treating AA and to guide physicians in making rational drug choices.

## Data Availability

The original contributions presented in the study are included in the article/supplementary material. Further inquiries can be directed to the corresponding author.
